# Outcomes and risk factors of SARS‐CoV‐2 omicron variant in B‐cell lymphoma patients following CD19 targeted CAR‐T therapy

**DOI:** 10.1002/cam4.6657

**Published:** 2023-11-14

**Authors:** Xibin Xiao, Panpan Chen, Yadi Zhong, Xiu Luo, Yao Liu, Ying Lu, Xueli Jin, Wenbin Qian, Weidong Han, Aibin Liang, Hui Liu

**Affiliations:** ^1^ Department of Hematology, The Second Affiliated Hospital, College of Medicine Zhejiang University Hangzhou China; ^2^ Department of Bio‐Therapeutic The First Medical Centre, Chinese People's Liberation Army General Hospital Beijing China; ^3^ Department of Hematology, Tongji Hospital Tongji University School of Medicine Shanghai China; ^4^ Department of Hematology Oncology Chongqing University Cancer Hospital Chongqing China; ^5^ Department of Hematology, Yinzhou Hospital, Affiliated to College of Medicine Ningbo University Ningbo China

**Keywords:** B‐cell lymphoma, chimeric antigen receptor T cell, omicron variant, outcome, severe acute respiratory syndrome coronavirus 2

## Abstract

**Background:**

Little was known on infection and mortality rates, still less the risk factors of severe acute respiratory syndrome coronavirus 2 (SARS‐CoV‐2) omicron variant in B‐cell lymphoma patients following CD19 targeted chimeric antigen receptor T cell (CAR‐T).

**Aims:**

We performed a retrospective multicenter study and analyzed the details of relapsed/refractory (R/R) B‐cell lymphoma patients who received CD19 targeted CAR‐T heretofore in five cellular immunotherapy centers in China during the omicron wave.

**Materials & Methods:**

One hundred fifty‐four patients were enrolled in this study.

**Results:**

Among them, 52 patients (33.8%) were uninfected, 74 patients (48.1) had ambulatory mild disease (including nine patients of asymptomatic infection), 22 patients (14.3%) had moderate disease and six patients (3.9%) had severe disease when data collected up. Three patients with severe disease died from COVID‐19, the death rate was 1.9% for all enrolled patients, and 2.9% for infected patients. We also found that patients over 60 years old or with diabetes mellitus (DM) tend to develop severe disease (*p* = 0.0057 and *p* = 0.0497, respectively). Patients had CAR‐T infusion within 6 months also tend to have severe disease (*p* = 0.0011). In multivariate logistic regression model, CAR‐T infusion within 6 months (relative risk (RR) 40.92; confidence interval (CI) 4.03–415.89; *p* = 0.002) were associated with significantly higher risk of severe disease.

**Conclusion:**

Through this study, we conclude that the outcome for B‐cell lymphoma patients following CD19 targeted CAR‐T therapy when facing omicron infection was improved, but aggressive precautionary measures were particularly crucial for patients with high risk factors.

## Graphical abstract

The outcome for B‐cell lymphoma patients following CD19 targeted CAR‐T when facing omicron infection was improved. But precautionary measures were crucial for patients who had CAR‐T infusion within 6 months.

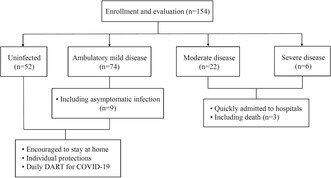

## 1 INTRODUCTION

Severe acute respiratory syndrome coronavirus 2 (SARS‐CoV‐2), which caused coronavirus disease 2019 (COVID‐19), has become a global public health emergency.^1^ As the pandemic progressed, SARS‐CoV‐2 has evolved from the initial alpha strain to the current omicron variant, characterized by decreased lung infectivity, less severe disease but higher transmissibility and capability of evading vaccinal immunity.^2^, ^3^, ^4^, ^5^, ^6^, ^7^ Therefore, from December 2022, the Chinese government declared to withdraw from “Zero COVID” policy when facing the omicron wave (mainly BA.5.2 and BF.7) of COVID‐19 pandemic.^8^, ^9^ And we are currently facing EG.5.1 globally.

Previous data showed that patients with immunodeficiency, especially hematological malignancies, tended to develop severe COVID‐19 with higher mortality in pre‐omicron era, which was attributed to primary diseases or anti‐tumor treatment procedures.^10^, ^11^, ^12^ As an important medical progress, chimeric antigen receptor T cell (CAR‐T) therapy targeting CD19 has shown great potential for patients with relapsed/refractory (R/R) B‐cell lymphoma.^13^, ^14^, ^15^, ^16^ So far, two CAR‐T products, relmacabtagene autoleucel (relma‐cel) and axicabtagene ciloleucel (axi‐cel), were approved by National Medical Products Administration (NMPA). Furthermore, CAR‐T clinical trials have been flourished and exhibited encouraging preliminary efficacy. In certain cases, CAR‐T might be the only option for patients in terminal phase.^13^, ^16^, ^17^ Unfortunately, this promising treatment was also accompanied with B‐cell aplasia, hypoglobulinemia, prolonged cytopenia, and invalid vaccination in particular.^13^, ^18^ Preliminary data showed that the vast majority of B‐cell lymphoma patients receiving CD19 targeted CAR‐T had very poor vaccine responses.^18^, ^19^

But with omicron variant, an EPICOVIDEHA survey reported a significantly lower death rate of 7% compared to 58% of the previous variants.^20^ Otherwise, little was known on infection and mortality rates, still less the risk factors, not to mention that our patients generally had no COVID‐19 history due to “Zero COVID” policy. Hence, in this paper we performed a retrospective multicenter study and analyzed the details of R/R B‐cell lymphoma patients who received CD19 targeted CAR‐T heretofore in five cellular immunotherapy centers in China during the omicron wave, trying to provide suggestions on CAR‐T whole‐course management for COVID‐19.

## 2 PATIENTS AND METHODS

### 2.1 Patients

We enrolled R/R B‐cell lymphoma patients who received CAR‐T therapy targeting CD19, still alive and in follow‐up on December 1, 2022, both from clinical trials or with commercial approvals, heretofore since April 2017 in four cellular immunotherapy centers in China, including the Second Affiliated Hospital, College of Medicine, Zhejiang University, Tongji Hospital of Tongji University, Chinese People's Liberation Army General Hospital, Yinzhou Hospital, Affiliated to College of Medicine, Ningbo University, and Chongqing University Cancer Hospital. The study was approved by the Ethics Committee of the Second Affiliated Hospital, College of Medicine, Zhejiang University, and each participating institution. The written informed consent was obtained from each patient included in this study or his or her agent and witness. Patients were quickly admitted to hospitals if they met the criteria of moderate or severe illness according to World Health Organization (WHO) clinical progression scale of COVID‐19 infection.^21^ Patients with uninfected or mild disease, including asymptomatic disease, were encouraged to stay at home, apply individual protections, and daily direct antigen rapid test (DART) for COVID‐19.^22^

### 2.2 Diagnosis and evaluation of lymphoma

Response to CAR‐T therapy was assessed by positron emission tomography‐computed tomography (PET‐CT) at the third month according to the International Working Group Response Criteria for Malignant Lymphoma.^23^ And duration of response was evaluated by peripheral blood (PB) minimal residual disease (MRD) screening via flow cytometry, ultrasonic or computed tomography (CT), as appropriate, every 3 months thereafter. These processes had been described in detail in our previous research.^13^, ^16^

### 2.3 Diagnosis of COVID‐19 and data recording

To make the diagnosis of COVID‐19 for patients who met the criteria of moderate or severe illness, two RNA isolation, and detection kits (Biogerm or Zhijiang, Shanghai, China) were used to extract SARS‐CoV‐2 RNA from nasal swabbed specimens, and real‐time reverse transcriptase‐polymerase chain reaction (RT‐PCR) was performed according to the manufacturer's instructions.^24^ For patients with uninfected or mild disease, DART was performed daily at home using the COVID‐19 antigen test kits approved by NMPA, according to the manufacturer's instructions and actual accessibility of the products.^22^

The disease history including previous treatments for B‐cell lymphoma, smoking status, comorbidities of the enrolled patients were obtained from electronic medical records and following‐up, as long as laboratory or imaging data. The COVID‐19 antigen test or nucleic acid test result, symptoms, signs, antiviral therapies, and supportive therapies were also carefully collected and evaluated by our trained team of hematologist. All clinical data were collected up to March 1, 2023. The disease severity was defined as the most severe stage during follow‐up.

### 2.4 Statistical analysis

IBM SPSS version 26.0 (IBM, USA) was used for statistical analysis. Descriptive analyses of categorical parameters were expressed as numbers of patients and percentages. The Fisher exact test was performed for categorical data. Multivariate logistic regression models were used to determine the independent risk factors for severe COVID‐19, the stepwise selection was used to identify significant variables at a significance level of 0.05. For all analyses, a two‐sided *p* value of less than 0.05 was considered statistical significance.

## 3 RESULTS

### 3.1 Clinical characteristics of enrolled patients

**FIGURE 1:**
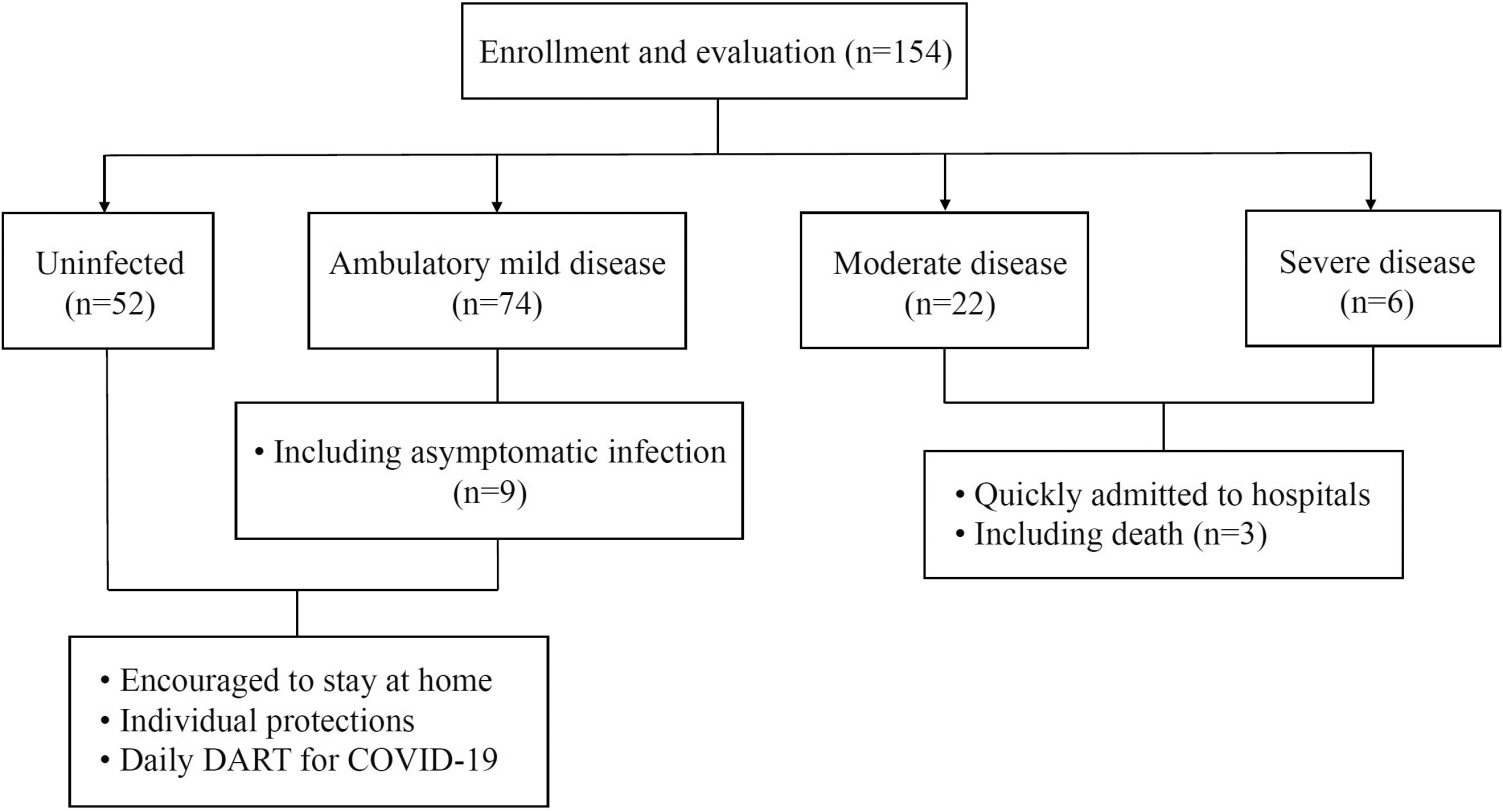
Study flowchart for 154 R/R B‐cell lymphoma patients who received CAR‐T therapy targeting CD19.

One hundred fifty‐four R/R B‐cell lymphoma patients who received CAR‐T therapy targeting CD19, still alive and in follow‐up, were enrolled in this study and presented in Figure 1, the median duration between CAR‐T infusion and study enrollment was 1183 days (2–2168 days). Due to China's previous “Zero COVID” policy, all patients had not been infected with COVID‐19 before enrollment. On December 8, 2022, the first patient was diagnosed with COVID‐19. Of these patients, 52 patients (33.8%) were uninfected, 74 patients (48.1) had ambulatory mild disease (including nine patients without any symptoms, namely asymptomatic infection), 22 patients (14.3%) had moderate disease and six patients (3.9%) had severe disease when data collected up. Unfortunately, despite our comprehensive treatments, three patients with severe disease died from COVID‐19. The death rate was 1.9% for all enrolled patients, and 2.9% for infected patients, which was much lower than preliminary data.^20^, ^25^ The clinical characteristics of patients were presented in Table 1. Notably, we enrolled patients still alive and in follow‐up, so we had 130 complete response (CR) patients (84.4%), three partial response (PR) patients (1.9%), eight patients (5.2%) with stable disease (SD) and 10 patients (6.5%) with progressive disease (PD) at the time of data collecting‐up. No patient with CR or PR experienced disease progression during following‐up. Three patients (1.9%) were not evaluated (NE), one of them died before PET‐CT evaluation at 3 months of CAR‐T infusion, other two patients were delayed because of COVID‐19 infection and had not been evaluated by PET‐CT until March 1, 2023.

**TABLE 1:** Clinical characteristics of enrolled patients.

Baseline characteristic	Number of patients	Percentage (%)
Sex		
Male	82	53.2
Female	72	46.8
Age^a^, years		
≤60	102	66.2
>60	52	33.8
Diagnosis^b^		
DLBCL	105	68.2
FL	16	10.4
MCL	13	8.4
PMBL	4	2.6
TFL	8	5.2
BL	4	2.6
CLL	2	1.3
RS	2	1.3
B symptoms^b^		
B symptoms	43	27.9
No B symptoms	111	72.1
ECOG Performance Score^b^		
0	62	40.3
1	76	49.4
2	13	8.4
3	3	1.9
Disease stage^b^		
I	5	3.2
II	21	13.6
III	38	24.7
IV	90	58.4
Extranodal sites^b^		
None	72	46.8
≥1	82	53.2
LDH^b^		
Normal	80	51.9
High	74	48.1
COVID‐19 infection		
Uninfected	52	33.8
Ambulatory mild disease	74	48.1
Moderate disease	22	14.3
Severe disease	6	3.9
Smoking status^a^		
Current smoker	7	4.5
Former smoker	29	18.8
Non‐smoker	118	76.6
Lymphoma status^b^		
Refractory	74	48.1
Relapsed	80	51.9
CAR‐T targeting type		
Targeting CD19	90	58.4
Dual targeting CD19 and CD20	64	41.6
CAR‐T infusion time		
Within 6 months	16	10.4
Over 6 months	138	89.6
Previous chemotherapy, lines^b^		
1	6	3.9
2	46	29.9
3	38	24.7
>3	64	41.6
Previous ASCT^b^		
Yes	23	14.9
None	131	85.1
Reponse status^a^		
CR	130	84.4
PR	3	1.9
SD	8	5.2
PD	10	6.5
NE^c^	3	1.9
Maintenance treatment^a^		
Yes	16	10.4
No	138	89.6
COVID‐19 vaccine status^a^		
None	141	91.6
1 dose	3	1.9
2 doses	5	3.2
3 doses	5	3.2

*Note*: Data were expressed as numbers of patients and percentages. The Fisher exact test was performed for categorical data.

Abbreviations: ASCT, autologous hematopoietic stem cell transplantation; BL, Burkitt's lymphoma; CAR‐T, chimeric antigen receptor T cell; CLL, chronic lymphocytic leukemia; COVID‐19, coronavirus disease 2019; CR, complete response; DLBCL, diffuse large B‐cell lymphoma; ECOG, Eastern Cooperative oncology Group; FL, follicular lymphoma; LDH, lactate dehydrogenase; MCL, mantle cell lymphoma; NE, not evaluated; PD, progressive disease; PMBL, primary mediastinal B‐cell lymphoma; PR, partial response; RS, Richter syndrome; SD, stable disease; TFL, transformed follicular lymphoma.

^a^ These data were collected at the time of study enrollment.

^b^ These data were collected at the time of CAR‐T infusion.

^c^ One patient died before PET‐CT at 3 months of CAR‐T infusion, other two patients were delayed because of COVID‐19 infection.

### 3.2 Risk factors for patients with severe COVID‐19

The median age of enrolled patients was 55.5 years, 53 (range 18–86) and 68 (range 56–76) for non‐severe and severe patients, respectively. In six patients with severe disease, five patients were over 60 years old, who tend to develop severe disease (*p* = 0.017). Patients with diabetes mellitus (DM) tend to develop severe disease (*p* = 0.0497). Interestingly, if we define age over 60 years old, active cancer other than lymphoma, chronic kidney disease (CKD), chronic obstructive pulmonary disease (COPD), body mass index (BMI) ≥30, serious heart condition and DM as risk factors, patients with at least one risk factor tend to have severe disease (*p* = 0.0071). There was no significant relationship between severe disease and B‐cell lymphoma related risk factors, such as subtypes, B symptoms, Eastern Cooperative oncology Group (ECOG) performance status, disease stage, extranodal involvement, lactate dehydrogenase (LDH) level, previous response status, and previous autologous hematopoietic stem cell transplantation (ASCT) at the time of CAR‐T infusion, as well as prior use of bendamustine and Bruton's tyrosine kinase (BTK) inhibitors, the data were presented in Table 2.

**TABLE 2:** Correlation between clinical characteristics and severe disease.

Clinical characteristics	Non severe disease (*n* = 148)	Severe disease (*n* = 6)	*p*‐value
Female, *n* (%)	69 (46.6%)	3 (50%)	1.0000
Median age^a^ (range)	53 (18–86)	68 (56–76)	See Below
Diagnosis of DLBCL^b^, *n* (%)	100 (67.6%)	5 (83.3%)	0.6651
B symptoms^b^, *n* (%)	39 (26.4%)	4 (66.7%)	0.0516
ECOG 2–3^b^, *n* (%)	15 (10.1%)	1 (16.7%)	0.4882
Disease stage III–IV^b^, *n* (%)	122 (82.4%)	6 (100%)	0.5902
Extranodal sites ≥1^b^, *n* (%)	79 (53.4%)	3 (50%)	1.0000
Elevated LDH^b^, *n* (%)	69 (46.6%)	5 (83.3%)	0.1057
Previous chemotherapy lines >2^b^, *n* (%)	97 (65.5%)	5 (83.3%)	0.6643
Previous ASCT^b^, *n* (%)	21 (14.2%)	2 (33.3%)	0.2200
Lymphoma status relapsed^b^, *n* (%)	76 (51.4%)	4 (66.7%)	0.6827
Reponse CR^a^, *n* (%)	126 (85.1%)	4 (66.7%)	0.2355
Current smoker^a^, *n* (%)	7 (4.7%)	0 (0%)	1.0000
COVID‐19 vaccine at least one dose^b^, *n* (%)	13 (8.8%)	0 (0%)	1.0000
CAR‐T only target CD19, *n* (%)	85 (57.4%)	5 (83.3%)	0.4016
CAR‐T infusion within 6 months, *n* (%)	12 (8.1%)	4 (66.7%)	0.0011
Maintenance treatment^a^, *n* (%)	15 (10.1%)	1 (16.7%)	0.4882
Prior BTK inhibitor^a^, *n* (%)	37 (25.0%)	3 (50%)	0.1813
Prior bendamustine^a^, *n* (%)	7 (4.7)	1 (16.7%)	0.2780
Risk factors for severe COVID‐19^a^, *n* (%)			
At least one risk factor	63 (42.6%)	6 (100%)	0.0071
Age >60 years	47 (31.8%)	5 (83.3%)	0.0170
Active cancer	1 (0.7%)	0 (0%)	1.0000
CKD	3 (2.0%)	0 (0%)	1.0000
COPD	2 (1.4%)	1 (16.7%)	0.1121
BMI ≥30	12 (8.1%)	0 (0%)	1.0000
Serious heart condition	4 (2.7%)	0 (0%)	1.0000
DM	8 (5.4%)	2 (33.3%)	0.0497

Abbreviations: ASCT, autologous hematopoietic stem cell transplantation; BMI, body mass index; BTK, Bruton's tyrosine kinase; CAR‐T, chimeric antigen receptor T cell; CKD, chronic kidney disease; COPD, chronic obstructive pulmonary disease; COVID‐19, coronavirus disease 2019; CR, complete response; DM, diabetes mellitus; DLBCL, diffuse large B‐cell lymphoma; ECOG, Eastern Cooperative oncology Group; LDH, lactate dehydrogenase.

^a^ These data were collected at the time of study enrollment.

^b^ These data were collected at the time of CAR‐T infusion.

Interestingly, 16 patients enrolled had CAR‐T infusion within 6 months (after June 1, 2022), four among them had severe disease, illustrating the significant relationship between severe COVID‐19 and CAR‐T infusion within 6 months (*p* = 0.0011). In our previous CD19 targeted CAR‐T studies, circulating normal B cells could be detected by flow cytometry as early as 6 months after CAR‐T, which we believe can partially explain this significant relationship.^13^

One patient received CD19 targeted CAR‐T due to relapsed DLBCL 30 months ago and reached CR, but unfortunately, the patient relapsed 18 months ago, so the patient received a second time CD19 targeted CAR‐T infusion but only best response of PD had been reached and rapidly progressed again. For the next step, the patient received optimized tandem CD19/CD20 CAR‐T that can target CD19 and CD20 simultaneously, for the third time, 12 months ago, with best response of SD. Then the patient had salvage chemotherapies and survived with uncontrolled DLBCL. Despite receiving three infusions of CAR‐T as long as lymphodepletion chemotherapies, the patient only developed mild and self‐limited disease during this epidemic.

The association of clinical factors and severity of COVID‐19 was analyzed according to the multivariate logistic regression models. In full multivariate models, we assessed the interactions of age (>60), B symptoms presented, elevated LDH level, CAR‐T infusion time (within 6 months), previous ASCT and combined DM, the data were listed in Table S1. In this model, CAR‐T infusion within 6 months (relative risk [RR] 40.92; confidence interval [CI] 4.03–415.89; *p* = 0.002) were associated with significantly higher risk of severe disease.

### 3.3 DART, laboratory data and symptoms of enrolled patients

Fifty‐two patients were diagnosed with uninfected disease referred to the negative results of DART, and 74 patients had ambulatory mild disease (including nine patients with asymptomatic infection) with the positive results of DART. The median duration of positive DART lasted for 8 days (4–22 days).

**FIGURE 2:**
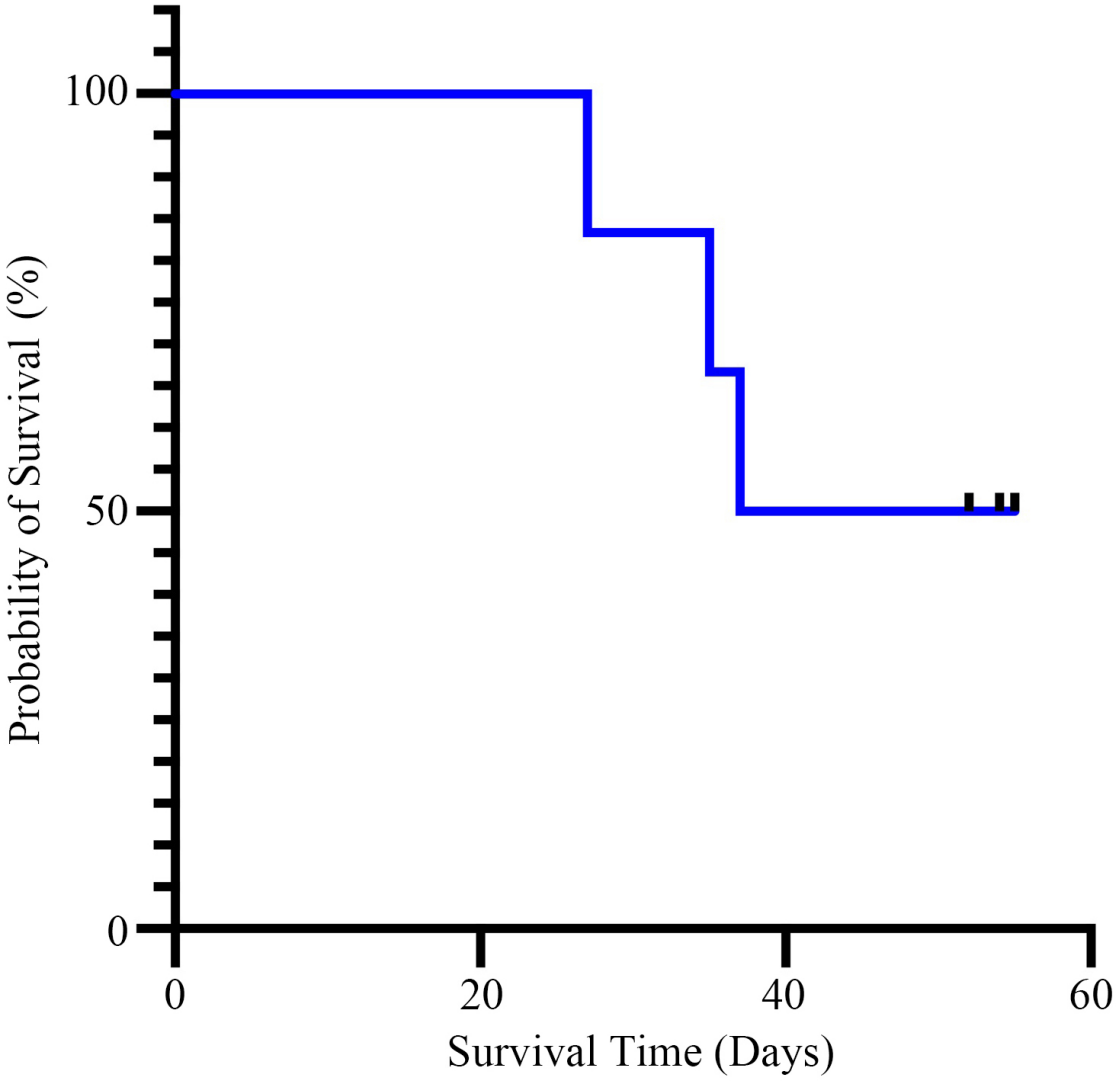
The Kaplan–Meier survival curve for these six patients with severe COVID‐19.

For the 22 patients with moderate disease, they were quickly admitted to hospitals. The duration between the onset of the symptoms and the nucleic acid test results turning negative or following up to time was 24 days (7–44 days), and four patients still had positive nucleic acid test results for 24 days, 38 days, 42 days, and 44 days until following up to time. For six patients with severe disease, they all admitted to intensive care unit (ICU), until following up to time or death, all six patients still had positive nucleic acid test results. The survival information for these six patients with severe COVID‐19 was presented with Kaplan–Meier survival curve in Figure 2.

## 4 DISCUSSION

Since CAR‐T therapy targeting CD19 had shown high efficiency and might be the only available option for certain patients, this novel treatment has become very important and definitive, especially during the COVID‐19 epidemic.^13^, ^14^, ^15^, ^16^ But to eliminate SARS‐CoV‐2 requires cooperative innate and adaptive antiviral responses, while immunocompromisation leads to underlying severe COVID‐19. Early data from EBMT Infectious Diseases Working Party and the European Hematology Association (EHA) Lymphoma Group showed that 39.3% patients with B‐cell malignancies after CAR‐T were admitted to ICU and 41.1% of them died from COVID‐19 infection during nearly half year observation, such outcome was extremely disappointing.^25^ As mentioned before, SARS‐CoV‐2 had gradually evolved to the latest omicron variant with new features. Recent data of lymphoma patients receiving CAR‐T therapy was still lacking. In this study, we described the COVID‐19 related details of R/R B‐cell lymphoma patients who received CD19 targeted CAR‐T heretofore in five cellular immunotherapy centers in China during the omicron wave, we found that only six patients (3.9%) suffered from severe COVID‐19, and three patients (1.9%) died from COVID‐19 infection or related complications in 3 months following‐up. This remarkable improvement might be partially due to our relatively shorter observation period, and more likely to the weakening of pathogenicity of omicron. The EPICOVIDEHA survey also reported the similar results.^20^

Preliminary results showed that patients with profound B‐cell aplasia following rituximab or other B‐cell targeted treatments were less likely to produce enough antibodies following COVID‐19 vaccination.^18^ So even though major population in China had been vaccinated with COVID‐19 vaccine, 141 patients (91.6%) were unvaccinated in our cohort. Only 13 patients were vaccinated with one or more doses, there were no severe disease occurred in these vaccinated patients. However, due to limited cohort volume, no significant difference was observed. Recent data showed that patients enjoyed normal or even heightened functional T‐cell responses, including anti‐ omicron T‐cell activities despite failed humoral response to mRNA‐based vaccines.^26^, ^27^ The EPICOVIDEHA survey also proved the efficiency of combined use of prior vaccination and monoclonal antibody treatment to reduce the risk of death.^20^ Collectively with our limited data, it is important to have COVID‐19 vaccination CAR‐T whole‐course management during pandemic.

Early data from EBMT Infectious Diseases Working Party and EHA Lymphoma Group showed patients with older age, worse performance status, metabolic comorbidities, and not being in CR at time of COVID‐19 diagnosis tended to have higher mortality risk.^25^ And in our cohort, patients over 60 years old incline to develop severe disease. If a patient has one of the following risk factors, including active cancer other than lymphoma, CKD, COPD, BMI ≥30, serious heart condition, or DM, he or she could be easily to have severe disease. Some of these risk factors had already been pointed out by previous studies.^10^, ^11^ Hematologists from Austria reported a case of successful CD19 targeted CAR‐T infusion in a DLBCL patient under severe COVID‐19 (omicron/BA.1) infection requiring mechanical ventilation after lymphodepletion therapy.^28^ But it was undeniable that in our cohort, CAR‐T infusion within 6 months were associated with significantly higher risk of severe disease in multivariate logistic regression models. Normal B‐cell recovery, improvement of agranulocytosis could partially explain this significant relationship.^13^ Aggressive precautionary measures were particularly crucial for these patients with high risk factors, such as vaccination, social distance and mask. Therefore, in our research design, patients with uninfected or mild disease, including asymptomatic disease, were encouraged to stay at home for safety concern.

It was reported that sensitivities of DART were 78.4% for participants within 5 days after the first RT‐PCR‐positive result and 90.77% even higher within 5 days after symptom onset in a Chinese cohort.^22^ And more importantly, nasopharyngeal swabs were better than oropharyngeal (throat) swab at sensitivity detection, especially in early stages of infection and in asymptomatic patients.^29^ So, in our research design, nasopharyngeal swabs were mandatory, both for DART and RT‐PCR, and DART results were approved for the diagnosis of COVID‐19.^30^

Comprehensive treatments were implemented for moderate or severe patients as soon as possible, including antiviral agents, intravenous immunoglobulin (IVIG), oxygen inhalation therapies, glucocorticoids, etc. Empirical antibiotics targeting bacteria and fungi were administered for patients with high possibility of related infections. The outcome for moderate patients was better than results from former studies, such that most patients recovered from COVID‐19 infection during following‐up.^25^ Interestingly, it was observed with a positive effect of convalescent plasma on survival for CAR‐T patients with COVID‐19. Unfortunately, no patient in our cohort received such a treatment.^25^

Our findings exhibited latest data, provided risk factors and underscored attention of COVID‐19 infection for patients under CD19 targeted CAR‐T during the omicron wave. Through this study, we conclude that the outcome for B‐cell lymphoma patients following CD19 targeted CAR‐T therapy when facing omicron infection was improved over time, but aggressive precautionary measures were particularly crucial for patients with high risk factors, such as the prevention of tixagevimab and cilgavimab, although currently not yet available in China.^31^ Nevertheless, our research still has some limitations. First, the cohort was relatively small. Second, out of safety concern, patients with uninfected or mild disease were encouraged to stay at home and DART was used for diagnosis. Third, important data such as dynamic monitoring of CAR‐T, B‐cell, and immunoglobulins were not fully obtained.

## AUTHOR CONTRIBUTIONS

**Xibin Xiao:** Data curation (equal); writing – original draft (equal). **Panpan Chen:** Formal analysis (equal); writing – original draft (equal). **Yadi Zhong:** Data curation (equal); investigation (equal). **Xiu Luo:** Data curation (equal); investigation (equal); project administration (equal); writing – original draft (equal). **Yao Liu:** Data curation (equal); formal analysis (equal); investigation (equal); writing – original draft (equal). **Ying Lu:** Data curation (equal); formal analysis (equal); investigation (equal); writing – review and editing (equal). **Xueli Jin:** Formal analysis (equal); funding acquisition (equal); writing – original draft (equal). **Wenbin Qian:** Investigation (equal); methodology (equal); project administration (equal); writing – review and editing (equal). **Weidong Han:** Formal analysis (equal); investigation (equal); project administration (equal); writing – review and editing (equal). **Aibin Liang:** Formal analysis (equal); investigation (equal); methodology (equal); writing – review and editing (equal). **Hui Liu:** Data curation (equal); investigation (equal); writing – original draft (lead).

## CONFLICT OF INTEREST STATEMENT

The authors declare no conflicts of interest.

## Supporting information


Table S1.

## Data Availability

For data sharing, contact the corresponding authors: hanwdrsw69@yahoo.com, lab7182@tongji.edu.cn, qianwb@zju.edu.cn, and sylen@zju.edu.cn.
