# Supplementary material for: Outcomes and risk factors of SARS‐CoV‐2 omicron variant in B‐cell lymphoma patients following CD19 targeted CAR‐T therapy
Source: Cancer Med. 2023 Nov 14;12(22):20838–46. doi: 10.1002/cam4.6657 (PMC10709723; doi:10.1002/cam4.6657)
Supplement: Supplementary file 1 — Table S1. [file CAM4-12-20838-s001.docx]

|  | RR | Coefficients | Standard Error | z | *P*-value | Lower 95% | Upper 95% |
| --- | --- | --- | --- | --- | --- | --- | --- |
| Intercept | 0.002073 | -6.1789 | 1.662 | -3.718 | 0.000 | 0.000080 | 0.053836 |
| Age>60 | 0.114376 | -2.1683 | 1.530 | -1.417 | 0.156 | 0.005701 | 2.294563 |
| B Symptoms | 2.947259 | 1.0809 | 1.081 | 1.000 | 0.317 | 0.354096 | 24.531027 |
| Elevated LDH | 7.481044 | 2.0124 | 1.379 | 1.460 | 0.144 | 0.501761 | 111.539184 |
| CAR-T Infusion Within 6 Months | 40.921014 | 3.7116 | 1.183 | 3.137 | 0.002 | 4.026410 | 415.886451 |
| Previous ASCT | 2.462933 | 0.9014 | 1.291 | 0.698 | 0.485 | 0.196280 | 30.904989 |
| DM | 5.878916 | 1.7714 | 1.441 | 1.229 | 0.219 | 0.349088 | 99.005457 |

**Supplementary Table 1.** The association of clinical factors and severity of COVID-19 according to the multivariate logistic regression models.

COVID-19, coronavirus disease 2019. RR, relative risk. CAR-T, chimeric antigen receptor T cell. LDH, lactate dehydrogenase. ASCT, autologous hematopoietic stem cell transplantation. DM, diabetes mellitus.
